# Expression of Beta-Defensin 131 Promotes an Innate Immune Response in Human Prostate Epithelial Cells

**DOI:** 10.1371/journal.pone.0144776

**Published:** 2015-12-09

**Authors:** Jung Hoon Kim, Kyeoung-Hwa Kim, Hae Jong Kim, Jaehyouk Lee, Soon Chul Myung

**Affiliations:** 1 Department of Urology, KEPCO Medical Center, Seoul, Korea; 2 Research Institute for Translational System Biomics, Chung-Ang University College of Medicine, Seoul, Korea; 3 Department of Urology, Chung-Ang University College of Medicine, Seoul, Korea; University of Kansas Medical Center, UNITED STATES

## Abstract

Previously, using the Illumina HumanHT-12 microarray we found that β-defensin 131 (DEFB131), an antimicrobial peptide, is upregulated in the human prostate epithelial cell line RWPE-1 upon stimulation with lipoteichoic acid (LTA; a gram-positive bacterial component), than that in the untreated RWPE-1 cells. In the current study, we aimed to investigate the role of DEFB131 in RWPE-1 cells during bacterial infection. We examined the intracellular signaling pathways and nuclear responses in RWPE-1 cells that contribute to DEFB131 gene induction upon stimulation with LTA. Chromatin immunoprecipitation was performed to determine whether NF-κB directly binds to the DEFB131 promoter after LTA stimulation in RWPE-1 cells. We found that DEFB131 expression was induced by LTA stimulation through TLR2 and p38MAPK/NF-κB activation, which was evident in the phosphorylation of both p38MAPK and IκBα. We also found that SB203580 and Bay11-7082, inhibitors of p38MAPK and NF-κB, respectively, suppressed LTA-induced DEFB131 expression. The chromatin immunoprecipitation assay showed that NF-κB directly binds to the DEFB131 promoter, suggesting that NF-κB is a direct regulator, and is necessary for LTA-induced DEFB131 expression in RWPE-1 cells. Interestingly, with DEFB131 overexpression in RWPE-1 cells, the accumulation of mRNA and protein secretion of cytokines (IL-1α, IL-1β, IL-6, and IL-12α) and chemokines (CCL20, CCL22, and CXCL8) were significantly enhanced. In addition, DEFB131-transfected RWPE-1 cells markedly induced chemotactic activity in THP-1 monocytes. We concluded that DEFB131 induces cytokine and chemokine upregulation through the TLR2/NF-κB signaling pathway in RWPE-1 cells during bacterial infection and promotes an innate immune response.

## Introduction

Innate immunity is regarded as a primitive and universal host defense system. It is the first line of defense against invading pathogens and conserved products of microbial metabolism, such as peptidoglycan (PGN), lipoteichoic acid (LTA), lipopolysaccharides (LPS), zymosan, flagellin, lipoprotein, CpG-DNA, and nucleic acids [1]. The initial sensing of infection is mediated by germline-encoded pattern recognition receptors (PRRs). Four classes of PRRs have been identified: 1) Toll-like receptors (TLRs) that recognize ligands on extracellular pathogens and in intracellular endosomes and lysosomes, 2) C-type lectin receptors (CLRs) that recognize carbohydrates on microorganisms, 3) RIG-I-like receptors (RLRs) that recognize viruses, and 4) NOD-like receptors (NLRs) that function as cytoplasmic pathogen sensors [2].

Human β-defensins are cationic antimicrobial peptides with six conserved cysteine residues stabilized by three disulfide bonds. These peptides contribute to innate immune responses [3]. Unlike α-defensins, which are expressed in neutrophils and Paneth cells, β-defensins are expressed in epithelial cells. They exhibit bactericidal, fungicidal, and antiviral activity. RWPE-1 cells, a human prostate epithelial cell line, produce β-defensins when stimulated with various bacterial components, indicating that β-defensins may be an important immunomodulatory factor in prostatic function [4,5]. Upregulation of the β-defensin gene and peptide production in response to bacterial products and inflammatory stimuli, such as tumor necrosis factor alpha (TNF-α) and interleukin-1β (IL-1β), depends on the activation of the NF-κB, AP-1, JAK2, and STAT3 signaling pathways [6–8]. Additionally, β-defensins enhance cytokine and chemokine production such that each β-defensin induces a unique pattern of cytokine expression: interleukin 8 (IL-8) and monocyte chemoattractant protein 1 (MCP-1) were upregulated by human β-defensins 1, 2, and 3 (HBD1, 2, and 3), while IL-6 and IL-10 were induced more selectively [9,10]. Interestingly, β-defensins act as chemokines for immature dendritic cells and memory T cells, and, thus, they may serve as an important bridge between innate and adaptive immunity [11]. β-defensins are antimicrobial peptides of key interest to researchers because of this role [12].

Using bioinformatic approaches that were based on hidden Markov chain models linked to BLAST searches of the whole human genome, more than 28 β-defensin family members have been discovered [13]. With the exception of β-defensin 118 (DEFB118) and DEFB123, no functional studies on other members of the β-defensin family have been reported. In our previous study, using Illumina HumanHT-12 microarray analysis, DEFB131 was found to be highly expressed in LTA-stimulated RWPE-1 cells. There are only two reports regarding the chromosomal location of the DEFB131 gene and characterization of DEFB131 expression in human tissues [13,14]. Unlike most human β-defensins, which are located on chromosome 8, DEFB131 is located on chromosome 4 and is moderately expressed in the human small intestine, testes, and prostate [14]. At present, the function of DEFB131 is unknown. Thus, the aim of this study was to evaluate the role of DEFB131 in innate and adaptive immunity of the human prostate. We used RWPE-1 cells to examine the intracellular signaling pathways and nuclear responses contributing to DEFB131 gene induction upon LTA stimulation. We also determined the chemotactic effect of DEFB131 on human monocytes.

## Materials and Methods

### Reagents and antibodies

Lipoteichoic acid (LTA) was purchased from Sigma-Aldrich (St. Louis, MO, USA). The p38MAPK inhibitor SB203580 and NF-κB inhibitor Bay11-7082 were purchased from Calbiochem (San Diego, CA, USA). The rabbit anti-p38MAPK, rabbit anti-phospho-p38MAPK, rabbit anti-IκBα, rabbit anti-phospho-IκBα, rabbit anti-NF-κB p65 antibodies, and normal rabbit IgG were purchased from Cell Signaling (Beverly, MA, USA). The mouse anti-ACTB and rabbit NF-κB p65 antibodies were purchased from Santa Cruz Biotechnology (Dallas, TX, USA). The rabbit anti-TLR2 and rabbit anti-DEFB131 antibodies were purchased from Abcam (Cambridge, UK).

### Cultures of RWPE-1 and other cells

The immortalized human prostate epithelial cell line RWPE-1 was obtained from the American Type Culture Collection (ATCC; Manassas, VA, USA). RWPE-1 cells were cultured in Keratinocyte-SFM (serum-free medium; Invitrogen; Carlsbad, CA, USA) supplemented with 0.05 mg/mL bovine pituitary extract (BPE) and 5 ng/mL epidermal growth factor (EGF) in a humidified atmosphere containing 5% CO_2_. The cells were routinely passaged at 80–90% confluence.

The human prostate cancer cell lines PC-3 and DU-145 were obtained from ATCC (Manassas, VA, USA) and were maintained in RPMI 1640 (Invitrogen; Carlsbad, CA, USA), supplemented with penicillin/streptomycin (100 U/mL and 100 mg/mL, respectively) and 10% fetal bovine serum (Invitrogen), and kept at 37°C in a humidified atmosphere of 5% CO_2_. Cell passage was routinely performed with 0.25% trypsin.

### Knockdown of human TLR2 in RWPE-1

SureSilencing TLR2 shRNA plasmids with neomycin resistance were used to deplete human TLR2 expression. The shRNA plasmids for TLR2 knockdown were purchased from Qiagen (KH01808N; Venlo, Netherlands). This system utilizes four plasmids expressing shRNA sequences targeting different regions of the TLR2 ORF and a negative control plasmid expressing a scrambled shRNA sequence. RWPE-1 cells were cultured in 6-well plates to approximately 90% confluence in Keratinocyte-SFM supplemented with BPE and EGF. The cells were then transfected with either 4 μg of TLR2 shRNA plasmid or 4 μg of negative control shRNA plasmid using Lipofectamine 2000 (Invitrogen) in Opti-MEM media (Invitrogen) according to the manufacturer’s instructions. After 24 h, the transfection medium was replaced with Keratinocyte-SFM containing BPE and EGF. After an additional 24 h, RWPE-1 cells were cultured at a lower density in selection medium containing 400 μg/mL of G418. The G418 concentration was reduced to 200 μg/mL for one week for selection and was further reduced to 100 μg/mL for maintenance.

### Reverse transcription-polymerase chain reaction (RT-PCR)

Total RNA was isolated from RWPE-1 cells using an RNeasy Kit (Qiagen) according to the manufacturer’s instructions. Complementary DNA (cDNA) was synthesized from 2 μg of total RNA using 0.5 μg of oligo dT primers according to the first-strand synthesis protocol (Promega; Madison, WI, USA). PCR reaction conditions and primer sequences for genes analyzed in this study are listed in Table 1. PCR products were electrophoresed on a 1.5% agarose gel. Gels were photographed and analyzed using a Bio-Rad Molecular Imager Gel Doc XR+ System (Bio-Rad Laboratories; Hercules, CA, USA). Relative gene expression levels were normalized to ACTB expression. All experiments were performed three independent experiments.

**Table 1:** Primer sequences and RT-PCR conditions.

Gene	Accession numbers	Primer sequence	Annealing temperature	Product size
**DEFB131**	NM_001040448	For–TCCAGCCAGAAGCTTCATTT	65°C	158 bp
	NM_001040448	Rev–ACCACTTCTTTTGTCCGTCA	65°C	158 bp
**TLR2**	NM_003264	For–GATGCCTACTGGGTGGAGAA	67°C	393 bp
	NM_003264	Rev–CGCAGCTCTCAGATTTACCC	67°C	393 bp
**TLR6**	NM_006068	For–AGACCTACCGCTGAAAACCAA	62°C	225 bp
	NM_006068	Rev–ACTCACAATAGGATGGCAGGA	62°C	225 bp
**CD14**	NM_000591	For–CGTGGGCGACAGGGCGT	67°C	777 bp
	NM_000591	Rev–TAAAGGTGGGGCAAAGGGTT	67°C	777 bp
**ACTB**	NM_001101	For–CCATCGAGCACGCATCGTC	68°C	375 bp
	NM_001101	Rev–CTCGGTGAGGATCTTCATGA	68°C	375 bp

For, forward; Rev, reverse

### Quantitative real-time RT-PCR

To measure the amount of mRNA in RWPE-1 cells, quantitative real-time RT-PCR analysis was performed using Rotor-Gene Q (Qiagen). The primers used to amplify the selected genes were purchased from Qiagen (QuantiTect Primer Assay). The Rotor-Gene SYBR Green PCR Kit (Qiagen) was used for monitoring amplification, and the results were evaluated using Rotor-Gene Q series software. Reaction mixtures were set up in a total volume of 25 μL containing 1 μL of cDNA (diluted 1:100), 12.5 μL of SYBR Green PCR master mix (Qiagen), and 20 pmol of each gene-specific primer. The cycling conditions were: 95°C for 5 min; followed by 40 cycles at 95°C for 5 sec and 60°C for 10 sec with a single fluorescence measurement. Upon completion of the PCR, fluorescence was measured as the samples heated from 60°C to 95°C at a rate of 0.2°C/sec. The melting curves were used to identify nonspecific amplification products. Quantitation of gene amplification was performed by determining the cycle threshold (C_T_) based on the fluorescence detected within the geometric region of the semi-log amplification plot. Expression of each mRNA species was normalized to the expression of internal control genes, such as ACTB, GAPDH, and HPRT1. Relative quantities of target gene expression were determined using the comparative C_T_ method, and experiments were repeated at least three times using different sets of RWPE-1 cells.

### Western blot

Total protein extracts from RWPE-1 cells were prepared in a lysis buffer of 1.5% sodium dodecyl sulfate (SDS), 62.5 mM Tris-HCl (pH 6.8), 5 mM ethylenediaminetetraacetic acid (EDTA), 1% 2-mercaptoethanol, 1 mg/mL of antipain, 1 mg/mL of chymostatin, and 1 mg/mL of leupeptin (all from Sigma-Aldrich). Lysates were clarified by centrifugation and the supernatants were stored at −80°C. To analyze secreted proteins, cell culture media were collected and concentrated using Amicon Ultra-0.5 centrifugal filter devices with a cut-off of 3,000 nominal molecular weight limit (Amicon Ultra 3K; Millipore, Darmstadt, Germany). Equal volumes of concentrated cell culture media were separated using 15% SDS-PAGE and transferred onto a 0.2 μm polyvinylidene difluoride membrane (Amersham Biosciences; Amersham, UK). The constituent proteins of the epithelial cell extracts were separated by SDS-PAGE on a 10% separating gel and then transferred to a nitrocellulose membrane (Bio-Rad Laboratories). The membrane was blocked for 1 h in Tris-buffered saline-Tween [TBST; 0.2 M NaCl, 0.1% Tween-20, and 10 mM Tris (pH 7.4)] containing 5% non-fat dry milk. The blocked membranes were then incubated with rabbit anti-p38MAPK (1:1000), rabbit anti-phospho-p38MAPK (1:1000), rabbit anti-IκBα (1:1000), rabbit anti-phospho-IκBα (1:1000), rabbit anti-TLR2 (1:1000), rabbit anti-DEFB131 (1:1000), and mouse monoclonal anti-ACTB (1:2000) antibodies in TBST. After incubation, membranes were incubated with horseradish peroxidase-conjugated anti-rabbit IgG (1:2000; Cell Signaling) or anti-mouse IgG (1:2000; Cell Signaling) in TBST for 1 h at room temperature. After each step, the membranes were washed several times with TBST, and bound antibody was detected using an enhanced chemiluminescence detection system (Amersham Biosciences) according to the manufacturer’s instructions.

### Immunocytochemistry

RWPE-1 cells were grown on glass coverslips, fixed with 4% paraformaldehyde for 40 min, and then permeabilized with 0.1% Triton X-100 for 20 min at room temperature (RT). Cells were blocked with 3% bovine serum albumin (BSA; Sigma-Aldrich) for 2 h to block non-specific antibody binding and then incubated overnight at 4°C with antibodies against DEFB131. After washing, cells were incubated with FITC-conjugated anti-rabbit IgG (1:1000 dilution; Santa Cruz Biotechnology) for 1 h at RT, and DNA was counterstained with propidium iodide (PI; Sigma-Aldrich). Coverslips were mounted with Fluorescence Mounting Medium (DAKO). Fluorescence was detected by confocal laser microscopy (Zeiss LSM 510; Zeiss, Jena, Germany).

### Production of recombinant DEFB131

DEFB131 overexpression vectors were purchased from ORIGENE (RC218014; Rockville, MD, USA). The cells were transfected with either 4 μg of the DEFB131-DDK-Myc vector or 4 μg of the pCMV6 empty vector using Lipofectamine 2000 in Opti-MEM media according to the manufacturer’s instructions. After 24 h, the transfection medium was replaced with Keratinocyte-SFM containing BPE and EGF. After another 24 h, RWPE-1 cells were plated at a lower density and cultured in selection medium containing 400 μg/mL of G418. The G418 concentration was reduced to 200 μg/mL for one week for selection and then further reduced to 100 μg/mL for maintenance. The cells were lysed and sonicated. The supernatants obtained from the cultured RWPE-1 cells transfected with the pCMV6 empty vector or DEFB131-DDK-Myc were concentrated using a 3-kDa molecular mass cut-off filter (Millipore) and stored at −80°C. Production of recombinant DEFB131-DDK-Myc protein was confirmed by western blot using an anti-Myc antibody following the manufacturer’s instructions.

### ELISA

Cytokines and chemokines were measured using the Human Inflammatory Cytokines Multi-Analyte ELISArray kit (Qiagen) and the Human Common Chemokines Multi-Analyte ELISArray Kit (Qiagen) according to the manufacturer's instructions. Briefly, 50 μL of medium was incubated in a 96-well plate for 1 h at room temperature. This was followed by several washing and incubation steps as follows: incubation with detection antibodies for 1 h; wash; incubation with avidin-horse radish peroxidase for 30 min; wash; color development for 15–30 min; development termination using stop solution; and colorimetric quantitation of cytokines or chemokines by reading the optical density at 450 nm. In addition, the concentration of DEFB131 was measured using a DEFB131 ELISA Kit (Uscn Life Science Inc., Wuhan, China) according to the manufacturer’s instructions. Serial dilutions of recombinant peptide provided by the kit were used for generating standard curves. The optical density of the wells was determined using a microplate reader (TECAN System Inc., Männedorf, Switzerland) set to 450 nm.

### Chromatin immunoprecipitation (ChIP)

Experiments were performed using a Chromatin Immunoprecipitation Assay Kit (Upstate, Lake Placid, NY, USA) according to the manufacturer's procedure. Briefly, 2 × 10^7^ cells were treated with 1% formaldehyde for 10 min at 37°C. Subsequent procedures were performed on ice in the presence of protease inhibitors. Cross-linked cells were harvested, washed with phosphate-buffered saline (PBS), and lysed in SDS lysis buffer (1% SDS, 10 mM EDTA, 50 mM Tris-HCl [pH 8.1]) for 10 min at 4°C. Chromatin was sonicated with five 10-sec pulses at 30% amplitude. After centrifugation, the supernatant was diluted 10-fold with chromatin immunoprecipitation dilution buffer (0.01% SDS, 1.1% Triton X-100, 1.2 mM EDTA, 16.7 mM Tris-HCl [pH 8.1], 167 mM NaCl). Diluted extracts were clarified in the presence of Protein G Agarose. One-tenth of the diluted extract was kept as an input for direct quantitative real-time PCR. The remaining extracts were incubated for 16 h at 4°C in the presence of 1 μg of anti-NF-κB p65 antibodies or normal rabbit IgG per mL, followed by a 1-h incubation with Protein G Agarose. Following extensive washing (details available upon request), bound DNA fragments were eluted by a 30-min incubation in elution buffer (1% SDS, 0.1 M NaHCO_3_). The DNA was recovered over a 4-h period at 65°C in elution buffer containing 200 mM NaCl. DNA was then incubated in the presence of Proteinase K (20 μg/mL) for 1 h at 45°C. DNA was extracted with phenol-chloroform and chloroform-isoamyl alcohol and was ethanol-precipitated before being subjected to real-time PCR.

### Monocyte chemotaxis assay

Chemotaxis was assayed in 24-well plates containing 5-μm (pore size) Transwell inserts (Corning Costar, Tewksbury, MA, USA). Briefly, THP-1 cells, a human monocytic cell line, were pre-incubated for 24 h in serum-free RPMI 1640 supplemented with 0.1% bovine serum albumin (Sigma-Aldrich). After starvation, these cells were washed twice in PBS and resuspended in RPMI 1640 containing 0.1 % BSA. For each well, 1 × 10^6^ THP-1 cells were placed in the upper chamber with a polycarbonate membrane at the bottom, and a chemoattractant suspension was added in the lower chamber. Chemoattractant suspensions consisted of 50-fold concentrated culture medium from pCMV6- (control) or DEFB131-DDK-Myc-transfected RWPE-1 cells. MCP-1 was used as a positive control in the assay because MCP-1 is a well-established chemotactic substance for monocytes. After incubation at 37°C for 24 h, the cells that migrated to the lower chamber of the Transwell were quantified using trypan blue dye exclusion. Data are expressed as the fold-increase in migration over that of cells transfected with the empty vector.

### Statistical analysis

Data were expressed as the mean ± standard error of the mean (SEM) and a *P*-value of < 0.05 was considered significant. Analysis of variance (ANOVA) and Student’s *t*-test were used to determine if significant differences between groups existed. Statistical analyses were performed using Microsoft Excel (Microsoft Co., Redmond, WA, USA) and PASW Statistics ver. 18.0 (SPSS Inc., Chicago, IL, USA).

## Results

### DEFB131 mRNA expression

**Fig 1:**
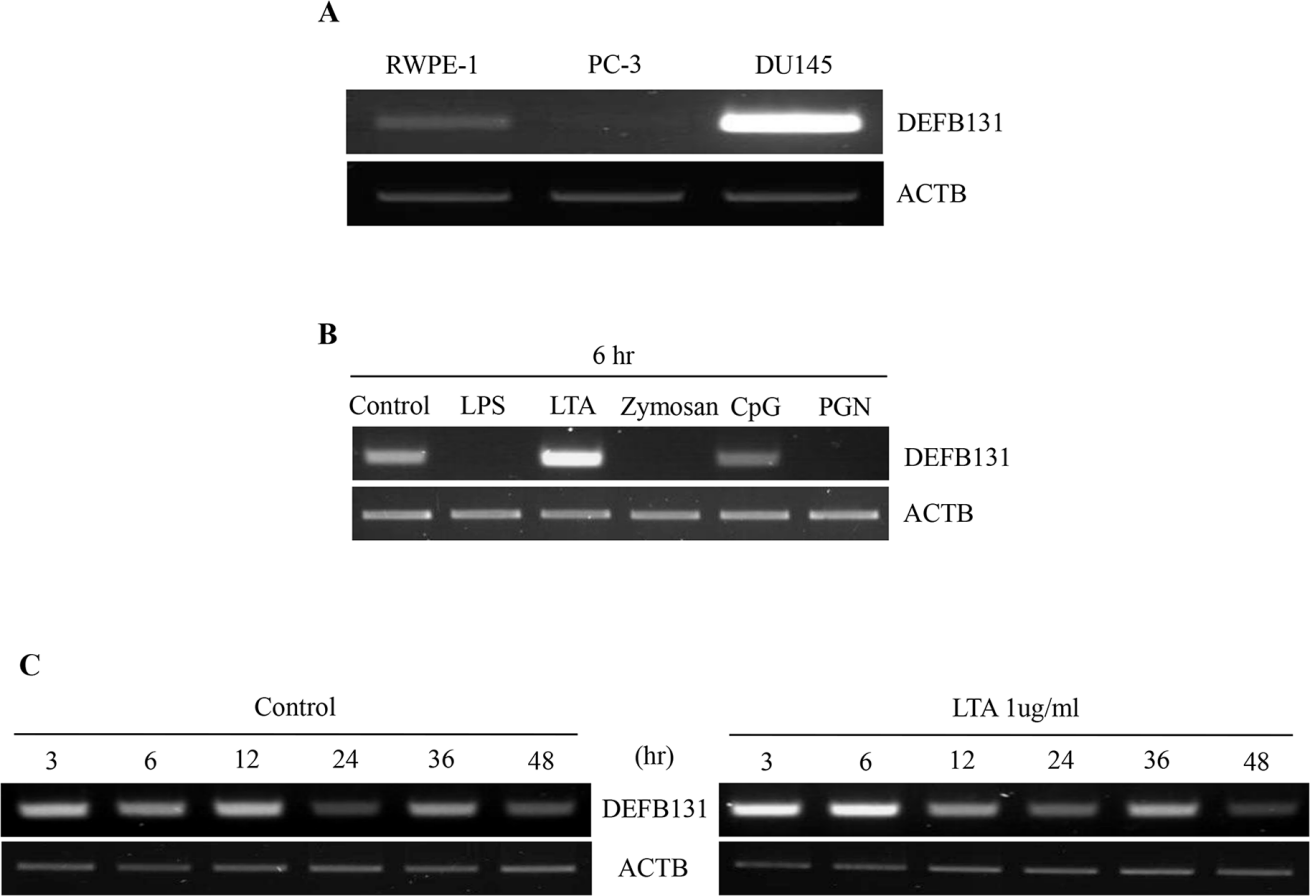
Expression of DEFB131 mRNA. **(A)** A semiquantitative RT-PCR analysis was performed on the immortalized human prostate epithelial cell line (RWPE-1) and the human prostate cancer cell lines (PC-3 and DU145). **(B)** Semiquantitative RT-PCR analysis of differential DEFB131 mRNA expression after RWPE-1 cell exposure to various TLR agonists. **(C)** Semiquantitative RT-PCR analysis of DEFB131 mRNA expression in RWPE-1 cells after treatment with LTA (1 μg/mL). ACTB was used as an internal control.

DEFB131 gene expression was examined in human epithelial cells lines using RT-PCR. DEFB131 expression was strongly upregulated in DU145 cells, only weakly expressed in RWPE-1 cells, and it was undetectable in PC-3 cells (Fig 1A). To confirm the microarray data, we performed an RT-PCR analysis on RWPE-1 cells stimulated with various TLR agonists. When RWPE-1 cells were treated with LTA for 6 h, DEFB131 expression was significantly increased (Fig 1B). In RWPE-1 cells, DEFB131 mRNA levels were increased at 3 h and markedly enhanced at 6 h after treatment with LTA (Fig 1C).

### LTA-induced DEFB131 expression via TLR2 in RWPE-1 cells

**Fig 2:**
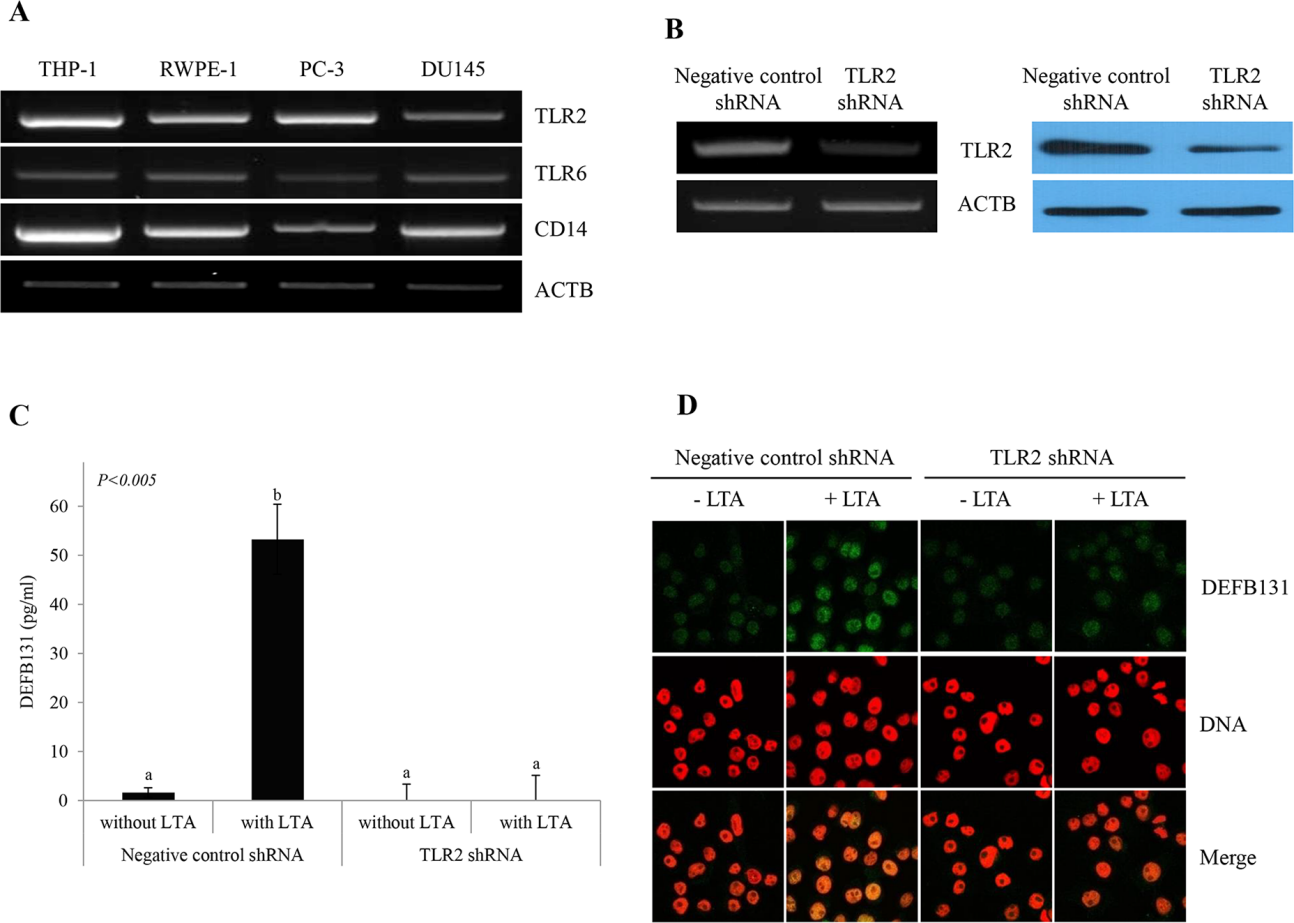
TLR2 is required for LTA-induced DEFB131 upregulation in RWPE-1 cells. **(A)** Semiquantitative RT-PCR analysis for differential expression of TLR2, TLR6, and CD14 in various human prostate cell lines. THP-1, a human monocyte cell line, was used as the positive control. ACTB was used as an internal control. **(B)** TLR2 gene expression in TLR2 shRNA-transfected RWPE-1 cells was determined using semiquantitative RT-PCR and western blot analyses. **(C)** LTA stimulates DEFB131 through TLR2 in RWPE-1 cells. The DEFB131 concentration was measured by ELISA. Concentrations are represented as pg of protein per mL of the supernatant and are representative of five distinct experiments. Different letters (a and b) indicate significant differences at *P* < 0.005 between groups. **(D)** An immunocytochemistry analysis was carried out to confirm the data obtained from the ELISA analysis. RWPE-1 cells were stained with anti-DEFB131 antibody (Green) and counterstained with propidium iodide (Red) for DNA.

CD14 mediates the TLR2/TLR6 heterodimer response to LTA. The expression of these genes in human prostate cells was evaluated by RT-PCR. Expression of TLR2, TLR6, and CD14 was detected in RWPE-1 cells, as well as in PC-3 and DU145 cells (Fig 2A). To determine whether LTA treatment led to the production of DEFB131 through TLR2, RWPE-1 cells were transfected with TLR2 shRNA to specifically knock down TLR2. RT-PCR and western blot analysis showed a marked decrease in TLR2 mRNA and protein expression following transfection of TLR2 shRNA (Fig 2B). We conducted ELISA and immunocytochemistry assays to determine the effects of TLR2 knockdown on the secretion of DEFB131 protein. When cells were stimulated with LTA, DEFB131 secretion was significantly decreased in TLR2-silenced RWPE-1 cells compared to negative control shRNA-transfected cells (Fig 2C and 2D). These findings indicated that LTA-induced DEFB131 expression was dramatically enhanced in the presence of TLR2.

### LTA-mediated p38MAPK and NF-κB activation induce DEFB131 gene expression

**Fig 3:**
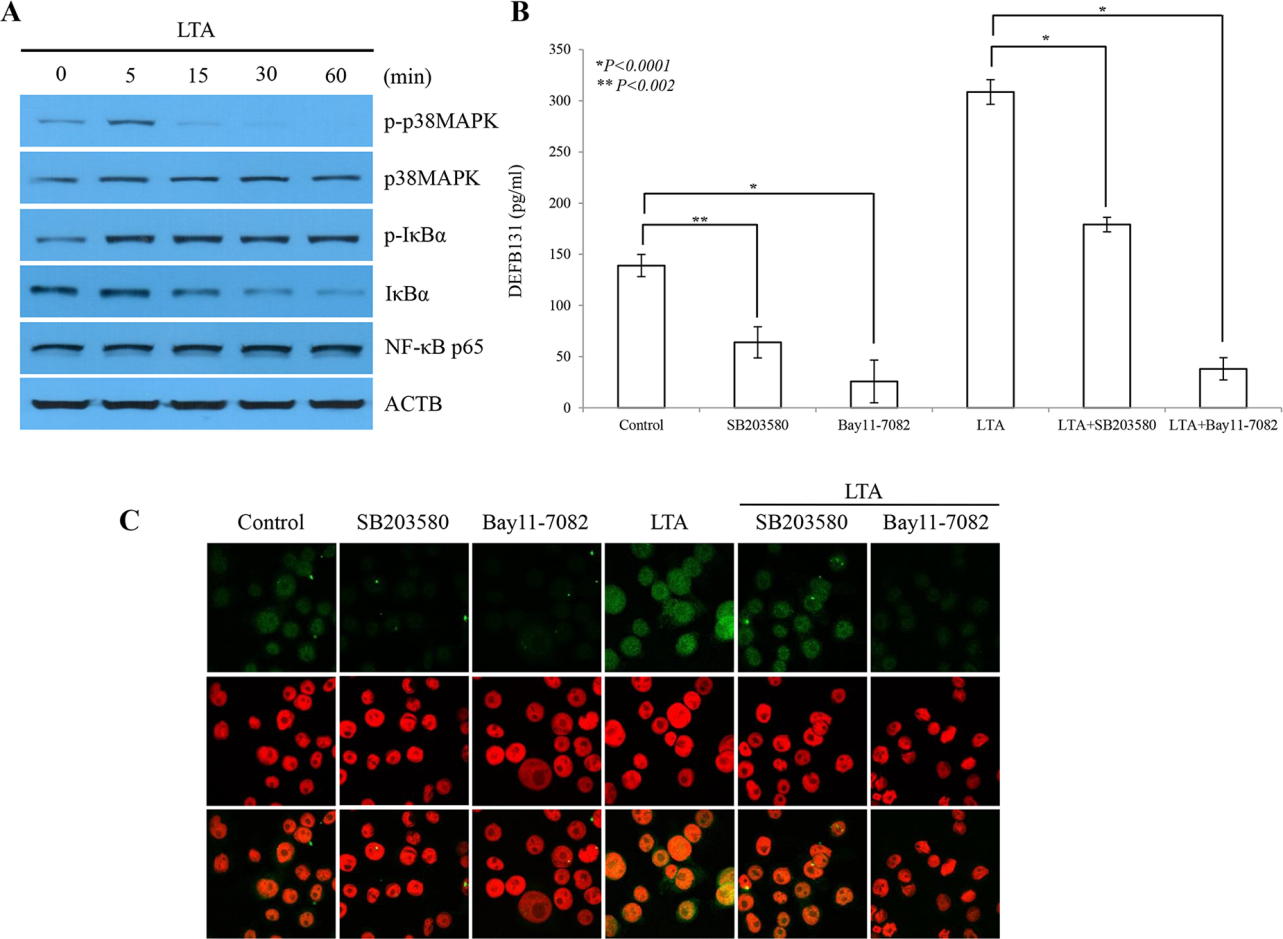
Induction of DEFB131 gene expression by LTA is p38MAPK- and NF-κB-dependent. **(A)** Phosphorylation of p38MAPK and IκBα determined by western blot after LTA stimulation. LTA stimulated an increase in p38MAPK and IκBα phosphorylation in RWPE-1 cells. In contrast, expression of IκBα was significantly reduced in a time-dependent manner. **(B)** Inhibition of p38MAPK and NF-κB blocks DEFB131 production. ELISA analysis showed that SB203580 (p38MAPK inhibitor) and Bay11-7082 (NF-κB inhibitor) inhibited secretion of LTA-induced DEFB131 protein. Asterisks (* and **) represent statistical significance at *P* < 0.0001 and *P* < 0.002. **(C)** Immunocytochemistry of DEFB131 after SB203580 or Bay11-7082 treatment showed a reduction in DEFB131 protein expression.

As shown in Fig 3A, western blotting revealed that IκBα expression gradually decreased between 5 and 60 min upon stimulation with LTA, whereas phosphorylated IκBα expression rapidly increased and was maintained within the same LTA exposure window (Fig 3A). Slightly higher levels of p38MAPK phosphorylation were detected after 5 min LTA stimulation, but thereafter phosphorylation was rapidly reduced. Western blot analysis showed that LTA activated both the p38MAPK and NF-κB signaling pathways. To further determine whether p38MAPK and NF-κB activation is required for LTA-mediated DEFB131 upregulation, we analyzed the effects of the p38MAPK inhibitor SB203580 and the NF-κB inhibitor Bay11-7082 on LTA-mediated DEFB131 protein expression. Protein levels were examined by ELISA and immunocytochemistry. High levels of DEFB131 protein expression were detected in LTA-stimulated RWPE-1 cells (Fig 3B and 3C). DEFB131 expression decreased after treatment with SB203580 or Bay11-7082. Taken together, these results suggested that p38MAPK and NF-κB activation induced LTA-mediated DEFB131 gene expression.

### The DEFB131 gene promoter has a functional NF-κB binding site

**Fig 4:**
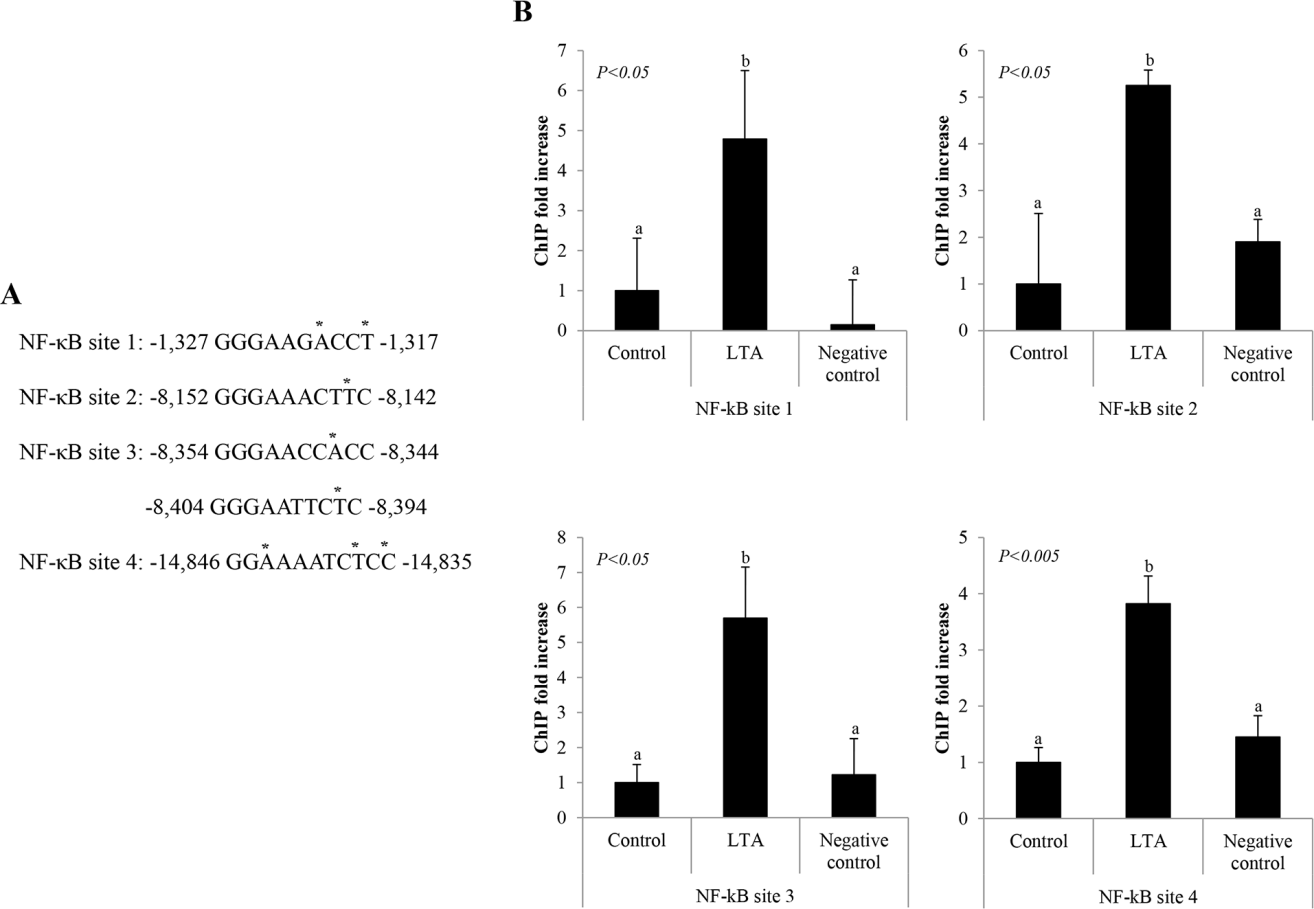
NF-κB binds to the DEFB131 gene promoter. **(A)** Five potential NF-κB/p65 binding sequences are located in the human DEFB131 promoter region. The differences in the site sequences from the consensus NF-κB binding sequences (CBS) are indicated by asterisks. CBS, GGGRNNYYCC or HGGARNYYCC; R, purine; Y, pyrimidine; H, A, C, or T. **(B)** Chromatin immunoprecipitation assays with antibody directed against NF-κB were performed on RWPE-1 cells treated with LTA. Different letters indicate significant differences between groups at *P* < 0.05 or *P* < 0.005.

To determine whether NF-κB directly binds to the DEFB131 promoter after LTA stimulation in RWPE-1 cells, we performed a ChIP assay. We identified five putative NF-κB binding sites in the DEFB131 gene using the TFSEARCH program (http://www.cbrc.jp/research/db/TFSEARCH.html). During sequence comparison, we observed that NF-κB binding sites in the DEFB131 gene were closely related to NF-κB consensus binding sequences (Fig 4A). To confirm an interaction between NF-κB/p65 (RelA) and the genomic DNA complex, DNA immunoprecipitated from LTA-treated and untreated RWPE-1 cells was analyzed by quantitative real-time PCR for sequences corresponding to the following NF-κB binding sites: −1,327 to −1,317 (NF-κB site 1), −8,152 to −8,142 (NF-κB site 2), −8,354 to −8,344 and/or −8,404 to −8,394 (NF-κB site 3), and −14,846 to −14,835 (NF-κB site 4) within the DEFB131 promoter. As expected, increased binding of NF-κB/p65 to the DEFB131 gene promoter region containing NF-κB sites 1–4 was observed (Fig 4B). This result provides clear evidence that NF-κB directly binds the DEFB131 promoter region at NF-κB binding sites in LTA-activated RWPE-1 cells, resulting in DEFB131 upregulation.

### DEFB131 enhances cytokine and chemokine production in RWPE-1 cells

**Fig 5:**
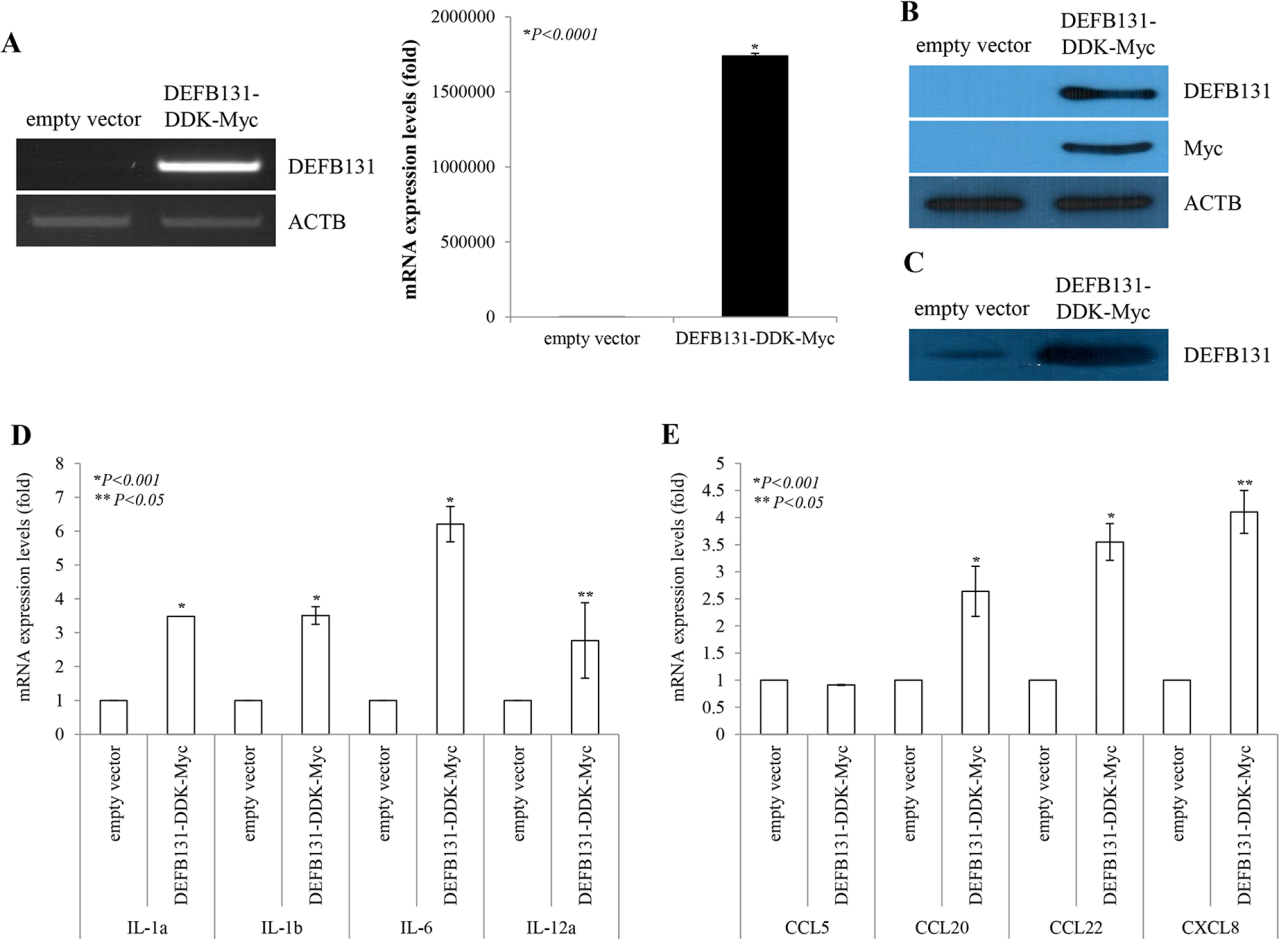
DEFB131 induces cytokine and chemokine expression in RWPE-1 cells. (**A**) DEFB131 mRNA upregulation. RWPE-1 cells were transfected with an empty vector or the DEFB131-DDK-Myc vector. Quantitative real-time PCR analysis was used to determine mRNA levels. Expression levels were calculated from C_T_ values and normalized to ACTB. Asterisks represent statistical significance at *P* < 0.0001. (**B**) Upregulation of DEFB131 protein. DEFB131 protein levels were analyzed by western blot in cell lysates. (**C**) Overexpression of secreted DEFB131 protein by western blot in culture supernatants. (**D, E**) Gene expression profiles for cytokines and chemokines in DEFB131-activated RWPE-1 cells were determined using quantitative real-time PCR analysis. Asterisks (* and **) represent statistical significance at *P* < 0.01 and *P* < 0.05.

**Fig 6:**
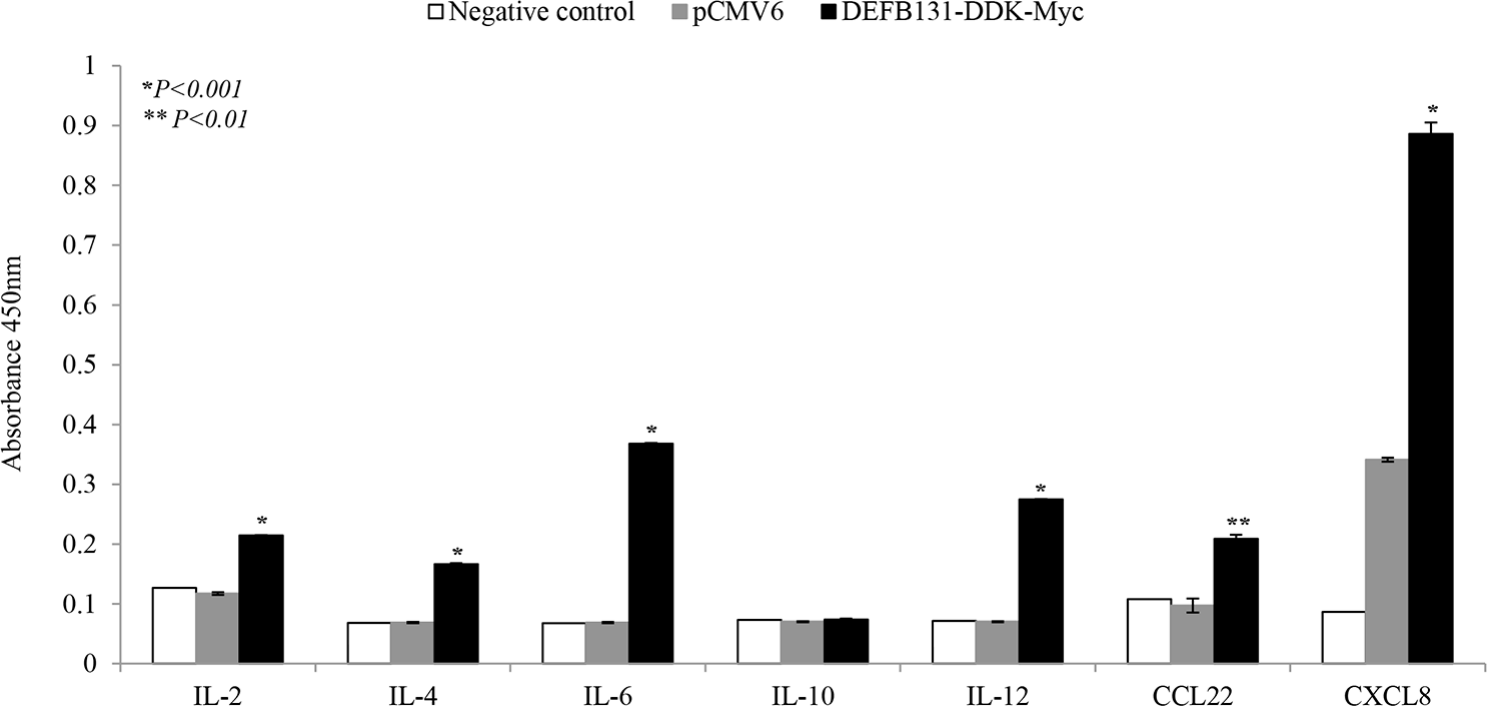
DEFB131 enhances cytokine and chemokine secretion in the culture supernatants of RWPE-1 cells. Supernatants obtained from RWPE-1 cells transfected with an empty vector or DEFB131-DDK-Myc were collected after 24 h. The levels of cytokines and chemokines released into the medium were quantified by multi-analyte ELISA. Error bars represent the standard error of the mean (SEM). Asterisks (* and **) represent statistical significance at *P* < 0.001 and *P* < 0.01.

To determine whether upregulation of DEFB131 leads to enhanced cytokine and chemokine production, RWPE-1 cells were transfected with the DEFB131-DDK-Myc plasmid, and then cytokine and chemokine expression levels were determined by quantitative real-time PCR. DEFB131 (mRNA, Fig 5A; non-secreted protein, Fig 5B; secreted protein, Fig 5C) was expressed at markedly higher levels in DEFB131-transfected RWPE-1 cells than in RWPE-1 cells transfected with an empty vector. Some cytokine and chemokine expression levels in the empty vector- and DEFB131-transfected RWPE-1 cells are shown in Fig 5D and 5E. Quantitative real-time PCR analysis revealed that expression of IL-1α, IL-1β, IL-6, IL-12α, CCL20, CCL22, and CXCL8 was significantly higher in DEFB131-transfected RWPE-1 cells than in empty vector-transfected RWPE-1 cells (Fig 5D and 5E). To confirm that increased cytokine and chemokine mRNA levels were associated with an increase in protein production, the protein levels of cytokines and chemokines secreted into culture supernatant were determined by ELISA. IL-6, IL-12, CCL22, and CXCL8 protein levels detected in the culture medium of DEFB131-transfected RWPE-1 cells were significantly higher than the levels in empty vector-transfected cells, consistent with the results of the quantitative real-time PCR analyses (Fig 6). Also, secreted IL-2 and IL-4 protein levels were increased in DEFB131-transfected RWPE-1 cells, whereas there was no change in IL-10 levels. These results indicated that upregulation of DEFB131 in RWPE-1 cells increased the production of proinflammatory cytokines and chemokines.

### DEFB131-induced chemokine and cytokine upregulation promotes monocyte chemotaxis

**Fig 7:**
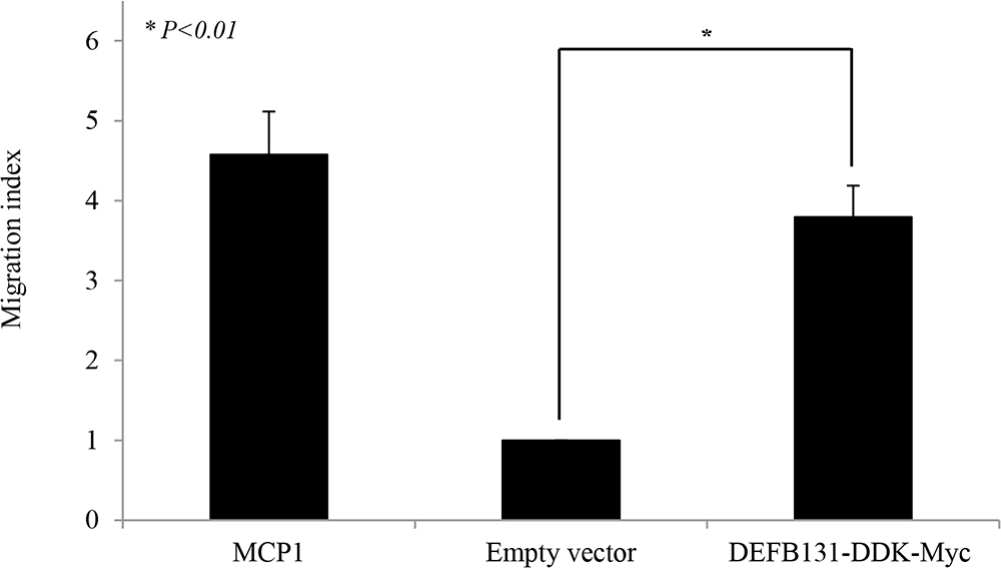
DEFB131 enhances chemotactic activity in monocytic THP-1 cells. Chemotactic activity was determined by measuring THP-1 migration in response to DEFB131-conditioned cell culture supernatant. The results are presented as a migration index denoting the fold-increase in cell migration as compared to the migration of cells transfected with the empty vector. MCP-1 (100 ng/mL) was used as a positive control. Results are representative of three independent experiments. Asterisks represent statistical significance at *P* < 0.01.

We used culture supernatants from RWPE-1 cells transfected with an empty vector as a negative control for the chemotaxis assay, whereas MCP-1 was used as the positive control for the THP-1 monocyte chemotactic activity assay. DEFB131-induced cytokines and chemokines were used as chemoattractants, and their ability to enhance THP-1 cell chemotaxis was determined. THP-1 monocytes were incubated with concentrated cell culture supernatants from DEFB131-transfected RWPE-1 cells for 24 h. As shown in Fig 7, culture supernatants from DEFB131-transfected RWPE-1 cells, containing DEFB131 and DEFB131-induced cytokines and chemokines, induced greater chemotactic activity in THP-1 cells (3.8-fold) than cell culture supernatants from empty vector-transfected cells. This indicated that enhanced chemotaxis was a result of cytokine and chemokine induction through DEFB131 upregulation.

## Discussion

Our previous study validated the use of microarray technology to investigate gene expression changes in RWPE-1 cells stimulated with various bacterial components. We compiled a list of genes in the human β-defensin family that were differentially expressed. This list was analyzed and sorted according to gene expression patterns. Among these genes, we selected DEFB131, located on human chromosome 4, for further analysis. In this study, we determined that DEFB131 expression in RWPE-1 cells was regulated by LTA via TLR2, and we demonstrated that DEFB131 upregulation enhanced cytokine and chemokine secretion through the NF-κB and p38MAPK signaling pathways, which in turn stimulated monocyte chemotaxis.

Human β-defensins are disulfide-stabilized cationic antimicrobial peptides that contribute to host defense responses against a broad spectrum of pathogens, including most bacteria, certain fungi, and enveloped viruses [3]. β-defensin peptides show marked differences in tissue expression, gene regulation, structural properties, and biological activities related to the human immune system [15]. To date, 34 genes in the human β-defensin family have been identified according to a conserved pattern of six cysteine residues [13]. Thus far, only a few of these β-defensins have been characterized at the protein level. One report determined by RT-PCR that DEFB131 was expressed in the small intestine, testes, and prostate. We also observed that LTA enhanced DEFB131 expression and secretion in the immortalized RWPE-1 prostate epithelial cell line (Figs 1 and 3).

Human β-defensin and TLRs are key elements that initiate innate immune responses against infection and influence the subsequent adaptive immune responses [3]. To date, most TLRs have been shown to recognize particular molecular structures of different pathogens [16]. More specifically, TLR4 recognizes LPS from gram-negative bacteria, TLR3 senses double-stranded viral RNA, TLR7 and TLR8 sense single-stranded viral RNA, TLR5 recognizes bacterial flagellin, and TLR9 recognizes bacterial CpG DNA. In contrast, TLR2 recognizes zymosan, a cell wall particle of yeast, as well as microbial products from gram-positive bacteria, such as LTA, PGN, lipoproteins, and mycobacterial cell wall components, indicating that TLR2 has a broader recognition profile [17–21]. This broad range of bacterial structures recognized by TLR2 may be explained by the ability of TLR2 to form heterodimers with other TLRs, such as TLR1 or TLR6 [22–24]. **TLR2**/**TLR6** heterodimers recognize LTA. Interestingly, TLR2 was reported to colocalize with TLR6 at the plasma membrane of monocytes [25]. CD14, a TLR2 coreceptor, was reported to mediate the response of the TLR2/TLR6 complex to LTA [26]. We observed that TLR2, TLR6, and CD14 mRNA were expressed in prostate epithelial cell lines as well as in prostate cancer cell lines (Fig 2A), and that these mRNAs were directly related to intracellular signaling upon stimulation with LTA in the human prostate. Moreover, our ELISA and immunocytochemistry data showed that TLR2-silenced RWPE-1 cells exhibited diminished DEFB131 production upon LTA treatment. Therefore, we concluded that DEFB131 expression and secretion was markedly increased through TLR2/TLR6 heterodimers. Furthermore, TLR2 may play an essential role in LTA-stimulated RWPE-1 cell responses, and it is directly regulated to DEFB131 expression.

Epithelial cells express TLR2 and demonstrate TLR2-dependent activation and nuclear translocation of p38MAPK and NF-κB that are required for upregulation of β-defensins and cytokines in response to TLR2 ligands such as LTA [27,28]. We provide evidence that LTA-induced DEFB131 gene expression in RWPE-1 cells is regulated by multiple TLR2-dependent pathways involved in the activation of p38MAPK and NF-κB signaling pathways (Fig 3) and the NF-κB gene transcription network in RWPE-1 cells (Fig 4). We also found that p38MAPK and IκBα are rapidly phosphorylated in LTA-treated RWPE-1 cells. Indeed, SB203580, a chemical inhibitor of p38MAPK, and Bay11-7082, a chemical inhibitor of NF-κB, significantly diminished DEFB131 production in LTA-stimulated RWPE-1 cells (Fig 3B and 3C). Therefore, we concluded that the p38MAPK and NF-κB pathways participate in LTA-mediated DEFB131 gene expression.

NF-κB plays a central role in innate immunity and inflammation, processes that contributes to β-defensin production. Activation of NF-κB via TLRs leads to the expression of antimicrobial peptides, cytokines, chemokines, inducible enzymes, and adhesion molecules that are important regulators or mediators of immunity. Wehkamp et al. (2004) performed luciferase gene reporter analyses and site-directed mutagenesis experiments and demonstrated that functional binding sites for NF-κB in the HBD2 proximal promoter are required for induction of HBD2, stimulated with *E*. *coli Nissle 1917*, in intestinal epithelial cells [29]. In the current study, we examined the DEFB131 promoter and identified functional NF-κB binding sites (positions −1,327 to −1,317, −8,152 to −8,142, −8,354 to −8,344 and/or −8,404 to −8,394, and −14,846 to −14,835), which are important for regulation of DEFB131 expression (Fig 4). It is noteworthy that DEFB131 expression is dependent on NF-κB activation.

Cytokines are messenger molecules that control the strength and duration of immune and inflammatory responses, while chemokines are a specific type of cytokine that directs migration of immune cells to infected cells. During an immune response against infection, both cytokines and chemokines are secreted by numerous immune and epithelial cells. This study showed that DEFB131 secretion is sufficient to stimulate immune pathways and induce expression of a wide range of proinflammatory cytokines and chemokines in RWPE-1 cells (Figs 5 and 6).

In many cases, when epithelial cells produce cytokines, their production levels may increase in response to other cytokines [30]. IL-1, a proinflammatory cytokine, is found in two main forms, α and β, that stimulate *de novo* production of IL-6 by epithelial cells [31,32]. Results showed that DEFB131-transfected RWPE-1 cells showed considerable upregulation of IL-1 α, IL-1 β, and IL-6 (Fig 5D). These cytokines are major mediators of innate immune reactions that share similar biological activities; they are also known as endogenous pyrogens [33]. These results suggested that fever, a part of the innate immune response, is due to DEFB131-induced endogenous pyrogens such as IL-1 α, IL-1 β, and IL-6. We also determined that IL-2 and IL-12 were highly expressed in DEFB131-transfected RWPE-1 cells (Figs 5D and 6). Previous research reported that IL-2 is a part of the IL-12 pathway in NK cells because IL-2 stimulates IL-12 receptor expression. This indicated that IL-2 is linked to IL-12 by signal transduction [34]. Both IL-2 and IL-12 are known to increase NK cells, and they enhance cytotoxic and cytolytic activities through the upregulation of perforin, cytolytic proteins, and granzyme genes in human NK cells [35]. Indeed, IL-12 exhibited anti-cancer and anti-metastatic activity with minimal toxicity. Thus, there is substantial evidence indicating that IL-2 and IL-12 are potent immunoregulatory cytokines, and, consequently, they are suspected to have potential therapeutic uses. IL-10, an anti-inflammatory cytokine, is known to inhibit the production of a number of proinflammatory cytokines, resulting in the inhibition of cell-mediated inflammation [36]. Interestingly, we observed no significant changes in IL-10 secretion in DEFB131-transfected RWPE-1 cells, which is consistent with the controls. These results support findings that epithelial cells produce greater than normal amounts of proinflammatory cytokines in diseased or infected tissues.

Chemokines act as chemoattractants to recruit immune cells to sites of inflammation. CXCL8, also known as IL-8, is a type of CXC chemokine involved in angiogenesis, which is important for tumorigenesis, cancer progression, and metastasis [37]. Furthermore, CXCL8 was shown to be a neutrophil and lymphocyte chemoattractant [38]. Our results showed that CXCL8 production in DEFB131-transfected RWPE-1 cells is markedly increased (Figs 5E and 6). Like IL-12, CXCL8 expression levels correlated with disease progression in several human carcinomas [37,39]. Chemokines of the CC chemokine family, such as CCL2, CCL12, and CCL22, are secreted proteins involved in immunoregulatory and inflammatory processes. These chemokines induce chemotactic activity in monocytes and other cell types, such as NK cells and dendritic cells, directing these cells toward sites of inflammation. Recent studies have shown that expression of CCL20, CCL22, and CXCL8 were significantly enhanced in DEFB131-induced RWPE-1 cells. Indeed, DEFB131-induced chemokines enhanced the chemotactic activity of THP-1 cells, which may be dependent on DEFB131-induced cytokines (Fig 7). These results indicated that high levels of proinflammatory cytokines (IL-1 α, IL-1 β, IL-2, IL-6, and IL-12) and chemokines (CCL20, CCL22, and CXCL8) in DEFB131-transfected RWPE-1 cells, and perhaps the high level of DEFB131 expression itself, may be associated with the recruitment of immune cells to sites of inflammation. We concluded that LTA-induced DEFB131 production plays an important role in mediating the release of proinflammatory cytokines and chemokines in RWPE-1 cells, and that this process may enhance inflammatory responses in prostate epithelial cells.

This study had a notable limitation. In some studies, commercially available LTA was found to contain endotoxin contaminants at potentially significant levels [40]. Endotoxin-containing LTA may serve as a TLR2 agonist and increase expression of DEFB131. For more accurate results, purification of the LTA reagent was needed. We did not perform a purification of commercially available LTA in this study. However, we performed some experiments on the expression of DEFB131 in the presence of endotoxin, LPS, in RWPE-1 cells. As a result, we found that DEFB131 expression was not increased by LPS.

## Conclusions

**Fig 8:**
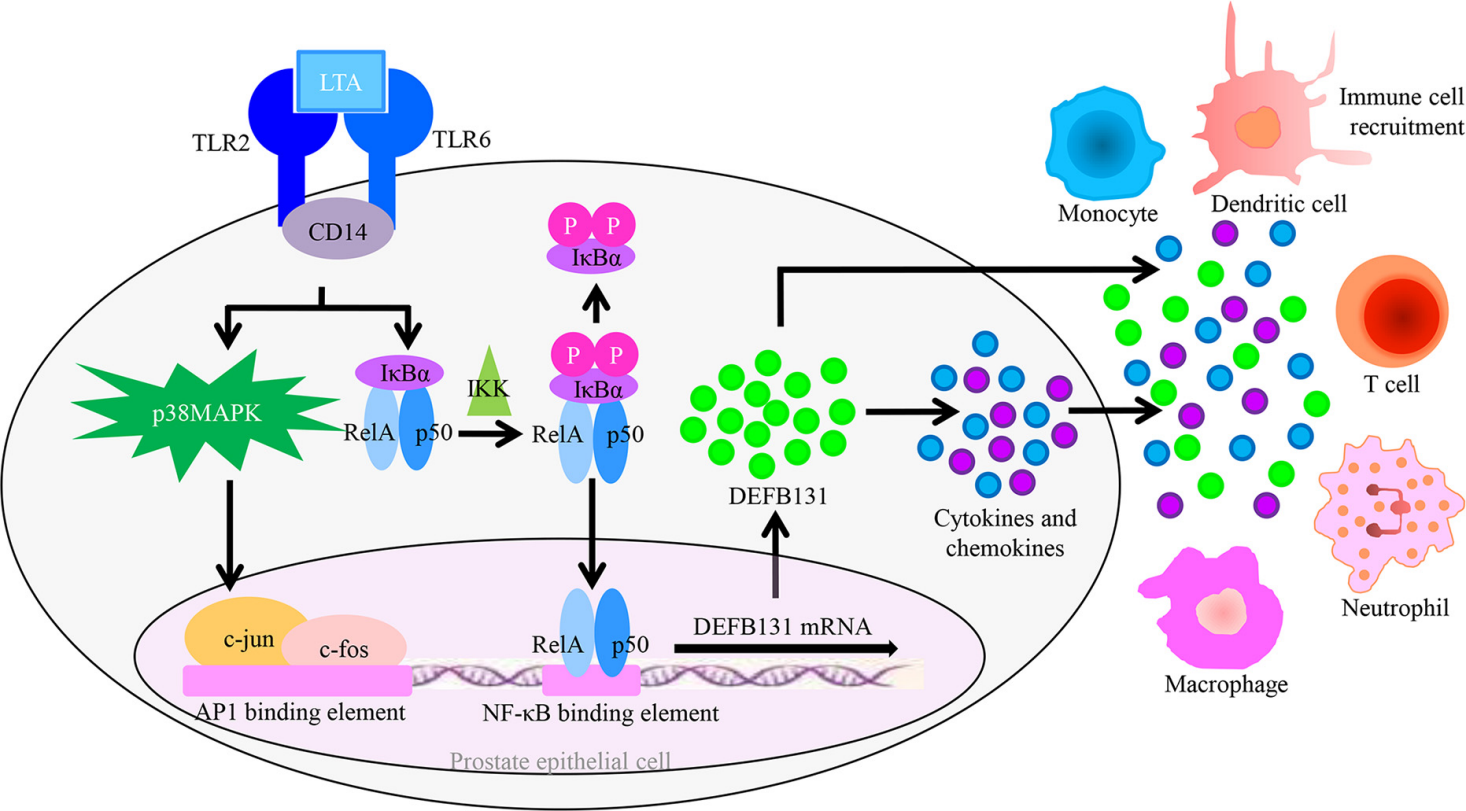
DEFB131 affects innate and adaptive immune responses in human prostate epithelial cells. LTA binds to the TLR2/TLR6 receptor complex and activates AP1 and NF-κB transcription factors through stimulation of the p38MAPK and NF-κB pathways in RWPE-1 cells. Upon LTA stimulation, the activated transcription of AP1 and NF-κB markedly induces DEFB131 expression and secretion, followed by increased production of cytokines and chemokines. Secreted DEFB131 exhibits antimicrobial activity. DEFB131 induces cytokine and chemokine expression and subsequent immune cell recruitment. DEFB131 may contribute to the activation of prostate epithelial cell innate and adaptive immune responses.

To our knowledge, this is the first study directly linking DEFB131 expression to cytokine and chemokine expression in human prostate innate immunity. Our data add to a framework for understanding LTA-induced DEFB131 expression in RWPE-1 cells as a part of the host defense reaction in the human prostate. Upon LTA stimulation, TLR2 recruits signaling molecules to intracellular signaling pathways within RWPE-1 cells, leading to p38MAPK and NF-κB activation. This results in the induction of an acute response and the expression and secretion of DEFB131, proinflammatory cytokines, and chemokines (Fig 8). This increase in DEFB131 expression may play a pivotal role in the mobilization of monocytes and the initiation of adaptive immune responses at sites of infection. Therefore, in conclusion, our results suggest a critical role for DEFB131 in innate and adaptive immune responses within the human prostate.
